# Compliance to Continuous Positive Airway Pressure therapy in patients with obstructive sleep apnea – long-term assessment

**DOI:** 10.5935/1984-0063.20200118

**Published:** 2021

**Authors:** Josefina Pascua, Magali Blanco, Glenda Ernst, Alejandro Salvado, Eduardo Enrique Borsini

**Affiliations:** 1 Hospital Británico de Buenos Aires, Sleep Unit - Buenos Aires - Ciudad Autónoma de Buenos AIres - Argentina.; 2 Hospital Británico de Buenos Aires, Center for Respiratory Medicine -Buenos Aires - Ciudad Autónoma de Buenos AIres - Argentina.; 3 Hospital Británico de Buenos Aires, Teaching and Research Department -Buenos Aires - Ciudad Autónoma de Buenos AIres - Argentina.

**Keywords:** Obstructive Sleep Apnea, CPAP Therapy, Compliance, Dropout

## Abstract

**Introduction:**

To assess CPAP acquirement, compliance, and dropout rates among OSA patients three years after the prescription.

**Material and Methods:**

We assessed CPAP acquirement (Acq), compliance (Comp), and dropout (Dout) through a telephone survey. We interviewed 156 patients; ESS: 9.9±5.7, AHI>15ev/hour in 96.4%. 92 patients had accessed CPAP therapy (58.9%) and 67 (72.8%) were still using it. Dropout was reported by 25 (27.2%). The Comp group was older (p<0.01), had more men (p<0.001), higher ESS scores (p<0.03) and a higher level of specialist follow-up (p<0.001). Multivariate analysis adjusted showed; follow-up by experts (OR: 4.39; p<0.05) and ESS>10 (OR: 1.25; p<0.05) increased CPAP compliance.

**Conclusion:**

There is a high number of patients without CPAP therapy acquirement. Long-term compliance was found in ¾ of the study population in symptomatic patients followed up by specialists. Finally, 43% had effective treatment.

## INTRODUCTION

The prevalence of obstructive sleep apnea (OSA) ranges between 9% and 38% in the general population^1, 2^ and it is close to 28% in Latin America^3^. Such data prove the need for practical strategies for almost one billion people worldwide^4^.

In our country, epidemiological data are based on estimates. According to the Guidelines of the Argentine Association of Respiratory Medicine^5^, there are more than 5 million OSA patients among the Argentine adult population. One third of them suffer from moderate-to-severe OSA and, therefore, would be candidates to continuous positive airway pressure (CPAP) therapy.

Therapy efficacy is conditioned by the correct diagnosis and treatment prescription. In the case of CPAP therapy, compliance rests on the adequacy of the device and factors like; insurance coverage, follow-up and patient’s tolerance. In this way, compliance to CPAP therapy has been associated with several factors, such as perceived severity and symptomatology^3^.

Argentine Diagnosis and Treatment Guidelines include coverage recommendations about the diagnostic procedures for all suspected OSA patients, as well as CPAP treatment for patients with a confirmed diagnosis^6^.

However, since OSA is a chronic disease, compliance also depends on financial and social factors, such as socioeconomic status, economic and financial barriers to therapy^7^.

Data published in our country reveal that barriers to CPAP therapy are related to lack of consensus in CPAP indication or treatment priorities among treating physicians and lack of health insurance coverage or financial resources^8, 9^.

According to Latin American reports, one-third of CPAP therapy candidates never start treatment^10^. A deeper understanding of the local problems faced by each organizational system could contribute to improve CPAP acquirement and prevent dropouts that raise risks to unacceptable levels. Thus, we decided to interview OSA patients 3 years after their indication of CPAP therapy.

## OBJECTIVES

To assess CPAP acquirement, compliance, and dropout rates among OSA patients with CPAP therapy indication for three years after the prescription.

## MATERIAL AND METHODS

### Study population

Prospective study performed in a sample of consecutive patients with diagnosis of OSA who underwent a CPAP titration study at the Respiratory Medicine Unit of Hospital Británico, in Buenos Aires, in 2016.

The study protocol was approved by the Ethics Committee and the Institutional Review Board in accordance with the ethical principles of the declaration of Helsinki as amended (protocol: CRIHB ? 985).

### Inclusion criteria

Patients over the age of 18-years-old;

Patients with diagnosis of OSA established through polysomnography (PSG) or respiratory polygraphy (RP);

Patients with an indication of CPAP therapy according to guidelines who underwent a CPAP titration study in 2016 (at least 36 months before the telephone survey).

### Exclusion criteria

Psychiatric disorders or impairments that interfere with CPAP therapy;

Refusal to take part in the survey.

### Clinical data

Data were obtained from a systematic collection database belonging to the sleep unit of a University Center. We analyzed clinical data using Epworth Sleepiness Scale (ESS) scores, baseline weight and body mass index (BMI) in kg/m^2^, severity according to baseline AHI, and CPAP titration efficacy reported by the titration study.

Treatment type and characteristics (type of device, heated humidifier, masks, and prescribed pressure) were obtained in the telephone interview and sleep unit medical records.

### Definitions

*Acquirement to CPAP* (*Acq*): patients who had obtained a CPAP device either on their own or through their health insurance provider.

*Dropout (Dout):* a =30-day interruption of CPAP therapy before the administration of the survey.

*Compliance* (*Comp*) group: patients using CPAP at the time of the survey.

### Telephone survey

The telephone survey was conducted by 3 interviewers in April 2019. An introductory explanatory text was read out to candidates who offered to take part in the survey voluntarily and anonymously. Patients gave their express oral consent to participate.

The standardized survey seeked information on CPAP acquirement and dropout by 2 questions: “Have you accessed CPAP therapy?” (*acquirement*) and “Are you still using CPAP therapy?” (*compliance*).

- Other data gathered included the following:
- Anthropometric data;
- Indication of treating physician(s);
- Perceived disease severity;
- Symptoms and evolution during treatment;
- Intolerance to the device;
- Reported compliance rate (hours/night and days/ week);
- Characteristics of follow-up;
- Potential dropout factors;
- Coverage type and percentage;
- Administrative difficulties.

### Statistical analysis

We used descriptive statistics to study the population and dropout and compliance variables. Qualitative variables were presented as absolute values and percentages. Quantitative variables with normal distribution were presented as mean values and standard deviation and quantitative variables without normal distribution as median values and percentiles (25-75%). Differences were compared using Fisher’s exact or ?^2^ test for qualitative variables and Mann-Whitney or student’s t-test for quantitative variables.

We performed a bivariate analysis to include variables in a logistic regression model. After obtaining statistically significant predictive variables, we conducted a multivariate analysis for compliance-dropout predictors: age, sex, perception of symptoms at the beginning of therapy, and type of follow-up. Statistical analysis was conducted using GraphPad Prism-6™ and Med Calc 12.7 software with the support of the teaching and research department.

## RESULTS

**Figure 1:**
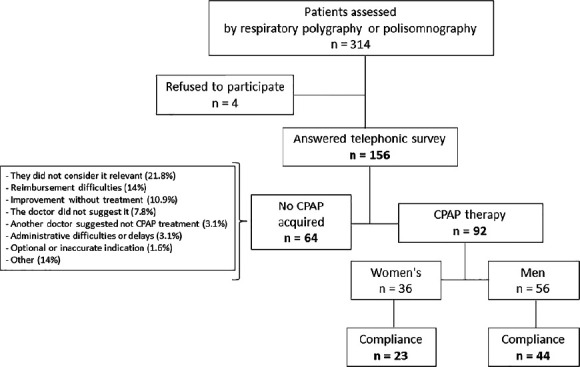
Patient selection flowchart.

Four patients refused to take part in the study. Finally, we interviewed 156 patients. Figure 1 shows patient selection. The mean time from diagnosis was 39.4 months. 101 were men (64.7%), BMI: 31.6±9.6 (kg/m^2^), baseline ESS: 9.9±5.7 points, baseline AHI: 33.2±19.0ev/hour and an obesity proportion of 80%. OSA was diagnosed by RP in 74.8% of cases and 96.4% were considered moderate-to-severe patients (AHI>15ev/h) (Table 1).

**Table 1:** Characteristics of study population (n=156).

Age (years)	64.6±10.8
BMI. (kg/m^2^)	31.6±9.6
Obesity (BMI>30 kg/m^2^)	125 (80.6%)
Sex (men)	101 (64.7%)
Diagnosed using respiratory polygraphy (n=%)	116 (74.8)
CPAP compliance during titration (min)	362±96.3
Baseline ESS score (points)	9.9±5.7
Baseline AHI (ev/h)	33.2±19.0
AHI>5 - <15ev/h (%)	3.6
AHI>15 - <30ev/h (%)	37.0

Notes: BMI = Body mass index; AHI = Apnea-hypopnea index; CPAP: Continuous positive airway pressure.

Treatment modality in compliant patients included fixed pressure CPAP (98.5%), heat humidifier (10.4%), nasal masks (67.2%), oronasal masks (28.3%), and pillows (4.5%). The mean treatment pressure was 8.4±1.7cm of H_2_O (Table 2).

**Table 2:** Treatment characteristics in both groups that used CPAP therapy.

	*Dropout (n=25)*	*Compliance (n=67)*
Fixed pressure CPAP therapy (%)	100	98.5
Auto-adjusting CPAP machine (%)	*	1.5
Heat-humidifier (%)	*	10.4
Prescribed pressure (cm H2O)	9.5±0.71	8.4±1.7
Time from the beginning of therapy (months)	24	39.4±28.7
Reported hours of use	*	6.4±1.4
Reported use (days per week)	*	6.5±1.2
Nasal pillows (%)	*	4.5
Nasal mask (%)	80	67.2
Oronasal mask (%)	20	28.3
Age of mask (months)	*	17.1±11.5
Attendance to CPAP education program (n; %)	3 (12)	16 (23.9)

*No data provided by patient; CPAP = Continuous positive airway pressure

Ninety-two (58.9%) patients acquired CPAP device and 67 (72.8%) of which maintained CPAP therapy and still using it at the time of the survey. The dropout rate was 25 patients (27.2%) with significant differences between sexes (36.1% of women and 21.4% of men) *p*<0.04.

Mean adherence in compliance group was 6.4±1.4 hours/night. There were statistically significant differences between *Comp* and *Dropout* groups. The *Comp* group was older (*p*<0.01), had more men (*p*<0.001), higher ESS scores (*p*<0.03), and a higher level of specialist follow-up (*p*<0.001) (Table 3).

**Table 3:** Characteristics of Dropout group (n=25) as compared to Compliant group (n=67).

Variable	A + B CPAP acquired (n=92)	A Dropout (n=5)	B Compliance (n=67)	p*
Age (years)	64.9 ± 10.5	58.3 ± 10.3	66.3 ± 10.6	0.01
BMI (kg/m2)	31.6 ± 9.5	31.2 ± 10.4	31.8 ± 9.7	0.81
Male sex (n=%)	56 (60.8%)	12 (48%)	44 (65.6%)	0.001
Initial CPAP compliance (min/night)	356 ± 96.3	329 ± 144	336 ± 102	0.27
Baseline ESS score (points)	9.9 ± 5.4	9.10 ± 6.8	12.5 ± 5.4	0.03
Baseline AHI (ev/hour)	27.4 ± 20.2	24.4 ± 19.0	28.6 ± 20.6	0.36
Specialist follow-up (n=%)		3 (12%)	46 (68.6%)	0.001

Notes: CPAP = Continuous positive airway pressure; BMI = Body mass index; AHI = Apnea-hypopnea index; *Statistical significance: p-value>0.05.

Sex, however, did not show useful discriminatory power as a predictor of dropout: odds ratio (OR) 1.95 (95% CI: 0.74-5.0), *p* 0.21 and AUC-ROC: 0.6. Reported dropout causes (n=25 cases) were treatment intolerance (16.6%), perceived clinical improvement (55.5%), and others (27.9%).

In the case of patients without acquirement to CPAP therapy (n=64), the main barriers were; lack of perception of disease relevance (21.8%), no medical indication (7.8%), disagreement in CPAP therapy recommendations (3.1%), improvement without treatment (10.9%), and difficulty in obtaining medical insurance coverage (14%) (Figure 1). Many CPAP devices were delivered with partial coverage by health insurance companies. Table 3 shows relationship between kind of therapy and mask with dropout.

More than half of the patients without acquirement to CPAP therapy stated that they had no other available treatment alternative. In fact, CPAP therapy was the only option offered to 80% of compliance group.

The multivariate analysis adjusted for sex and age showed that; follow-up by experts in respiratory medicine (OR: 4.39; *p*<0.05) and ESS>10 (OR: 1.25, 95% IC: 1.04-1.52, *p*<0.05) significantly increased patients’ probability of CPAP compliance (Table 4).

**Table 4:** Adjusted multivariate analysis and predictive model.

	OR	95% CI	p*
**Specialist follow-up**	4.39	1.5-12.75	<0.05*
**ESS (>10)**	1.25	1.04-1.52	<0.05*
**Age (>50 year)**	0.46	0.13-1.6	0.3
**Male sex**	0.74	0.1-5.0	0.7

Notes: OR = Odds ratio; 95% CI = Confidence interval.

## DISCUSSION

This analysis, conducted in OSA patients diagnosed according to standard guidelines who underwent a CPAP titration study, evidenced difficulties in acquirement and a dropout rate of 27% at the third year.

Only 43% of patients received effective treatment 39 months after CPAP indication, which revealed deficiencies in therapy initiation and follow-up processes. In our assessment, compliance according to sex differed from that reported in previous studies on chronic diseases^11^. Men with more symptoms (defined by a high ESS score) showed higher compliance to treatment.

In 2018, Nogueira et al.^10^ studied a sample of 213 patients with moderate-to-severe OSA (IAH>15ev/h) and an indication of CPAP therapy in the City of Buenos Aires. They found that 71% had accessed CPAP therapy but 15.5% dropped out before month 18. Though all patients had some kind of health insurance coverage, differences in access depended on coverage percentage: 59.2% received complete coverage and 49.2% partial coverage (*p*<0.001). Likewise, we found that one fourth of acquirement barriers related to coverage-related problems.

In the City of Mexico, Torres-Bouscoulet et al. (2007)^12^ described that 34.8% of patients did not access CPAP therapy after its indication. In a study conducted in Chile, approximately two thirds of OSA patients with an indication of CPAP therapy were still on treatment after a 12-month follow-up^13^. It is worth highlighting that 25% of Chilean patients who stopped using CPAP alleged financial reasons^13^. In spite of having different health systems in terms of organization and financing, other under developed countries in our region have reported similar difficulties in CPAP acquirement^10, 11, 12, 13^.

Argentina has a universal healthcare system and, therefore, at least in theory, all individuals should have access to health care. In addition, there are several jurisdictions and fragmented institutions which results in multiple decision-making centers around the country^14^, and the consequent delays and difficulties in treatment delivery.

In our data, reported monetary difficulties represent a modest percentage (14%) of cases. It is possible that others factors related with health organization could be responsible to did not get CPAP therapy, as administrative delays and unclear regulations.

Other potentially determining factors of acquirement and compliance are the methods used to deliver CPAP devices and follow-up patients. Local researchers have suggested that administrative delays and lack of communication among stakeholders undermine access and compliance to treatment^15, 16^. In this sense, there is evidence of an actual problem in the delivery of CPAP devices to patients^17^. A 5-center study conducted in the City of Buenos Aires evaluated 195 adult OSA patients with an indication of CPAP therapy. These devices were delivered by 22 different companies. Besides, significant differences in machine quality, technical support, and patient training and delay in administrative procedures. The actual delivery delay of CPAP devices was of 42.1±60.7 days.

OSA treatment efficacy does not depend solely on direct coverage. Rather, it calls for a strategic alliance among patients, patients’ families, healthcare centers, CPAP machine suppliers, and the community as a whole^15^.

In our setting, we have a specific education program for patients^8^, however, this strategy was neither mandatory nor binding, so attendance to our “CPAP school” was poor. Geographical and travel difficulties in large urban centers that limit the monitoring and education of patients could be complemented with telematics strategies or telemedicine.

The need to lower costs and increase treatment efficacy in these patients has promoted the development of remote surveillance to monitoring treatment using different tools^18, 19^. Remote surveillance with data transmission via Bluetooth, by phone, or using the internet is perceived as an alternative to improve compliance in the medium and long-term^20^, although the optimal organizational model and stakeholder roles in this model remain unclear^19^.

To the best of our knowledge, this is the first study conducted in our country that reports a long-term (>3 years) 72.8% compliance to CPAP. This information is of great value. We used a simple tool (i.e., telephone calls), as described by similar studies^15^, and found that, in line with findings from other under developed countries, more than half of the patients are not using CPAP therapy^11, 12, 13, 14, 15, 16, 17^.

A recent publication by the Argentine Society of Respiratory Medicine has pointed out the importance of treatment coverage and urged all stakeholders to discuss possible solutions addressing 3 levels: management, logistics, and administration and clinical aspect^10^.

Another drawback is the lack of alternatives other than CPAP therapy, as described by Nogueira et al. (2018)^10^. Stakeholders should discuss the possibility of having multiple treatments options and focus on educating the medical community and implementing organized healthcare policies^10^.

## CONCLUSION

According to our results, a large proportion of patients with clinically relevant OSA have no effective CPAP therapy despite confirmed diagnosis and correct titration. Long-term compliance is observed in three-fourths of the study population. Higher compliance is reported by more symptomatic patients and those followed up by specialists.
